# A validated workflow for paired total and small RNA sequencing from low-input submandibular gland biopsy specimens in *de novo* Parkinson’s disease patients

**DOI:** 10.3389/fnagi.2026.1931183

**Published:** 2026-08-31

**Authors:** Ko-Eun Choi, Joong-Seok Kim

**Affiliations:** 1 Department of Anatomy, Korea University College of Medicine, Seoul, Republic of Korea; 2 Department of Neurology, College of Medicine, The Catholic University of Korea, Seoul, Republic of Korea

**Keywords:** low-input tissue, Parkinson’s disease, small RNA sequencing, submandibular gland, transcriptomics

## Abstract

Parkinson’s disease (PD) is characterized by progressive α-synuclein aggregation, yet the molecular mechanisms underlying this process remain incompletely understood. Transcriptomic analyses may provide important insights into the RNA-mediated regulation of PD pathogenesis, but studies of affected brain tissue are limited by the inaccessibility of living brain tissue and post-mortem RNA degradation. The submandibular gland (SMG), which exhibits α-synuclein pathology comparable to that of the substantia nigra, represents a clinically accessible peripheral tissue for investigating disease-associated transcriptomic alterations. However, ultrasound-guided core needle biopsy yields only a limited amount of tissue, making comprehensive RNA sequencing technically challenging. Here, we present and validate a workflow for paired total RNA and small RNA sequencing from low-input SMG biopsy specimens obtained from patients with *de novo* PD and age-matched, neurologically unaffected surgical controls. Ultrasound-guided core needle biopsy yielded specimens measuring approximately 1.2 × 7–10 mm. To enable direct tissue-to-blood comparisons, fasting peripheral blood samples were collected from the same participants on the morning of biopsy. Following immediate tissue stabilization, RNA extraction, library preparation, and next-generation sequencing, workflow performance was evaluated by assessing RNA integrity, library quality, sequencing metrics, mapping performance, and reproducibility. Despite the limited tissue input, the protocol consistently generated high-quality RNA suitable for both total RNA sequencing and small RNA sequencing. The validated workflow produced robust sequencing libraries with high reproducibility and enabled comprehensive profiling of protein-coding transcripts together with multiple classes of small non-coding RNAs, including miRNAs, piRNAs, snoRNAs, snRNAs, and tRNA-derived RNAs. This validated workflow provides a practical and reproducible approach for comprehensive transcriptomic profiling of minimally invasive SMG biopsy specimens and matched fasting peripheral blood, and may facilitate future studies of disease mechanisms, biomarker discovery, and translational applications in Parkinson’s disease.

## Introduction

Aggregation of misfolded α-synuclein plays a crucial role in driving neural degeneration and the development of Parkinson’s disease (PD), while there is no disease-modifying treatment (Arias-Carrión, 2026). A central pathological hallmark of idiopathic PD is the accumulation of misfolded α-synuclein aggregates within vulnerable neuronal populations (Braak et al., 2003). Despite extensive study, the molecular mechanisms that drive the transition of α-synuclein from its soluble monomeric state to aggregated, pathogenic forms remain incompletely understood (Vidović and Rikalovic, 2022; Surguchov and Surguchev, 2022). While post-transcriptional (Martins et al., 2011) and post-translational (Beyer, 2006, Schmidt et al., 2022) mechanisms have been implicated, upstream or downstream regulatory factors that modulate α-synuclein biology are still being uncovered (Vidović and Rikalovic, 2022).

Growing evidence highlights the importance of RNA-based regulation in neurodegenerative diseases, including PD (Agarwal et al., 2020; Martirosyan et al., 2024; Choi et al., 2025). RNA metabolism—regulating mRNA stability, translation control, and the functions of non-coding RNAs such as microRNAs—has been shown to influence SNCA mRNA expression (Kattan et al., 2023; Doxakis, 2010; Rezaei et al., 2025). Because RNA can directly affect gene expression, protein synthesis, and pathways linked to protein folding and clearance, alterations in RNA networks may precede or exacerbate α-synuclein aggregation (Wang et al., 2025; Chiba-Falek and Nussbaum, 2001). However, the potential role of RNA in modulating α-synuclein misfolding remains underexplored, largely due to a lack of appropriately preserved tissues and comprehensive RNA profiling datasets.

Research on RNA dysregulation in PD has historically relied on post-mortem brain tissue (Agarwal et al., 2020; Martirosyan et al., 2024; Smajić et al., 2022; Kamath et al., 2022), where extended post-mortem intervals cause substantial RNA degradation (Dumitriu et al., 2012). These limitations complicate the identification of disease-associated RNA signatures and obscure early or subtle transcriptomic changes. Peripheral tissues that exhibit α-synuclein pathology in living patients therefore may offer a unique opportunity to investigate RNA-level alterations without the confounding effects of delayed tissue preservation (Choi et al., 2025). RNA profiling in such tissues may reveal early biomarkers and provide insights into mechanisms that drive α-synuclein dysregulation before widespread neurodegeneration occurs (Choi et al., 2025).

The submandibular gland (SMG) has emerged as a particularly relevant peripheral tissue for PD research (Chahine et al., 2020). The SMG is heavily innervated by autonomic fibers and consistently develops α-synuclein pathology comparable to that observed in the substantia nigra (Shin et al., 2019). Our previous transcriptomic analyses of salivary gland tissue have shown that the SMG captures disease-associated alterations in both coding and non-coding RNAs, with microRNA signatures in particular demonstrating superior sensitivity and specificity for identifying *de novo* PD (Choi et al., 2025). Because SMG tissue can be sampled through minimally invasive biopsy and does not suffer from the degradation issues inherent to post-mortem brain studies, it provides a high-integrity source for examining RNA regulatory mechanisms linked to α-synuclein aggregation (Choi et al., 2025).

Previous studies using submandibular gland (SMG) biopsy in Parkinson’s disease have primarily focused on the histopathological detection of phosphorylated α-synuclein for diagnostic purposes (Del Tredici et al., 2010; Beach et al., 2013; Chahine et al., 2020). More recently, transcriptomic analysis of SMG biopsy tissue highlighted the potential of this tissue as a molecular resource for investigating Parkinson’s disease in living patients (Choi et al., 2025). However, the application of SMG biopsy to transcriptomic studies presents substantial methodological challenges. Ultrasound-guided core needle biopsy requires accurate targeting to maximize tissue yield within a limited number of needle passes while minimizing patient discomfort and procedure-related complications. In addition, comprehensive transcriptomic profiling must be achieved from only a minute biopsy specimen (approximately 1.2 × 7–10 mm). Because transcriptomic analyses of such low-input SMG biopsy specimens remain limited, sharing a validated ultrasound-guided biopsy protocol together with quantitative information on RNA yield, RNA quality, library performance, and sequencing metrics may improve protocol standardization, reproducibility, and facilitate the broader adoption of SMG biopsy-based molecular studies in Parkinson’s disease.

Here, we present a validated workflow enabling paired total RNA and small RNA sequencing from low-input SMG core needle biopsy specimens obtained from patients with *de novo* PD and age-matched, neurologically unaffected surgical controls. Using biopsy specimens measuring approximately 1.2 × 7–10 mm, together with matched fasting peripheral blood collected on the morning of biopsy, we detail the complete workflow encompassing ultrasound-guided core needle biopsy, immediate tissue stabilization, optimized RNA extraction, standardized library preparation, and rigorous quality control for consistently generating high-quality RNA suitable for comprehensive transcriptomic profiling. This validated workflow provides a reproducible approach for comprehensive transcriptomic profiling of low-input SMG biopsy specimens and matched fasting peripheral blood, and may facilitate future molecular studies in Parkinson’s disease.

## Materials and methods

### Participants

**FIGURE 1:**
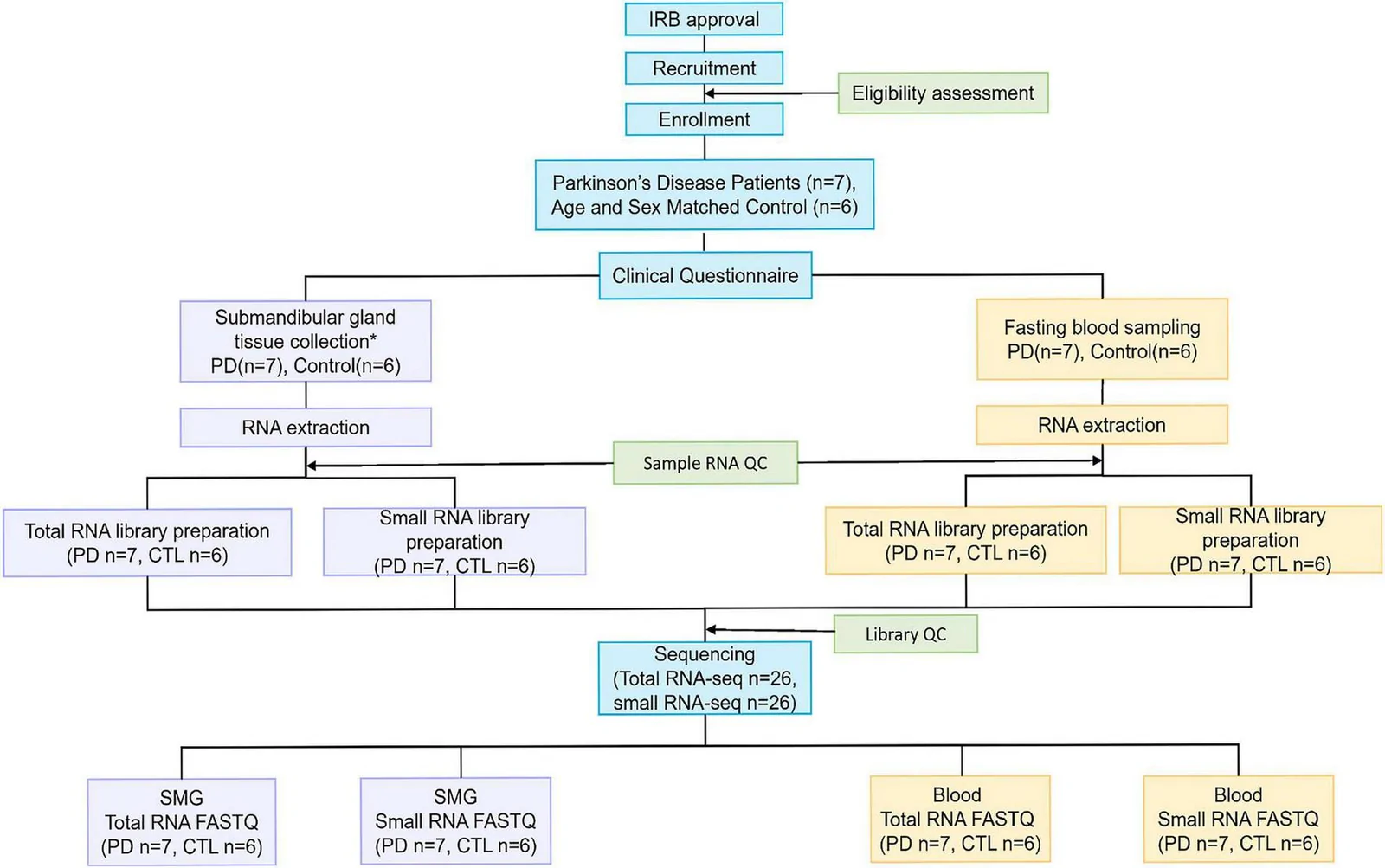
Overview of study workflow. The numbers of participants enrolled and ultimately included in the data analysis are shown in brackets. Blood sampling and SMG tissue collection were performed on the same day for each participant. * SMG tissue was obtained by ultrasound-guided core needle biopsy in the PD group, and by surgical excision in the control group. Tissue preservation interval (the time from tissue collection to RNAlater immersion) was < 1 min in PD patients and 1–20 min in controls.

The study workflow is summarized in Figure 1. The study cohort, previously reported by Choi et al. (2025), comprised seven patients with *de novo* Parkinson’s disease (PD) and six age- and sex-matched, neurologically unaffected surgical controls. PD was diagnosed independently by two board-certified neurologists at Seoul St. Mary’s Hospital according to the UK Brain Bank diagnostic criteria (Gibb and Lees, 1988). All patients were drug-naïve at enrollment and had not received levodopa treatment. Disease severity was assessed using the Hoehn and Yahr (H&Y) stage and the Unified Parkinson’s Disease Rating Scale Part III (UPDRS-III) at the time of diagnosis (Table 1).

**TABLE 1:** Demographic characteristics of patients.

Patient ID	Sex	Age	Smoking	Alcohol	UPDRS III	H&Y	Age at PD onset	PD duration at sampling (months)	LEDD
PD_donor1	M	56	0	1	52	2	55	17	0
PD_donor2	F	58	0	0	11	1	57	12	0
PD_donor3	M	65	1	1	3	0	64	12	0
PD_donor4	F	74	0	0	46	3	70	48	0
PD_donor5	M	67	1	0	30	2	66	12	0
PD_donor6	F	62	0	0	45	2	61	6	0
PD_donor7	M	67	1	1	26	3	66	12	0
CTL_donor1	F	64	0	0	NA	NA	NA	NA	NA
CTL_donor2	F	53	0	0	NA	NA	NA	NA	NA
CTL_donor3	M	67	0	0	NA	NA	NA	NA	NA
CTL_donor4	F	69	0	0	NA	NA	NA	NA	NA
CTL_donor5	M	58	1	0	NA	NA	NA	NA	NA
CTL_donor6	M	59	0	0	NA	NA	NA	NA	NA

UPDRS, Unified Parkinson’s Disease Rating Scale; H&Y, Hoehn and Yahr; LEDD, Levodopa Equivalent Daily Dose; NA, Not Applicable: Smoking/Alcohol 0 = no, 1 = yes.

Control SMG specimens were obtained from individuals who underwent submandibular gland resection for head and neck cancer between October 2022 and April 2023. Control participants underwent detailed neurological examination and medical record review to confirm the absence of neurological or psychiatric disorders, including Parkinson’s disease, essential tremor, idiopathic REM sleep behavior disorder, dementia, and depression. All participants were recruited through the Departments of Neurology and Otolaryngology–Head and Neck Surgery at Seoul St. Mary’s Hospital. Written informed consent was obtained from all participants prior to enrollment. Detailed clinical and pathological characteristics of the surgical control participants are provided in Supplementary Table 1.

Patients were excluded if they met any of the following criteria: (1) a family history of Parkinson’s disease; (2) early onset PD (symptom onset before 40 years of age); (3) clinical or neuroradiological findings suggestive of atypical or secondary parkinsonism; (4) the absence of presynaptic dopaminergic deficit on dopamine transporter imaging according to the Movement Disorder Society Clinical Diagnostic Criteria for Parkinson’s disease (Postuma et al., 2015); (5) structural abnormalities involving the basal ganglia or cerebellum on brain magnetic resonance imaging; (6) advanced dementia; (7) pre-existing submandibular gland pathology; (8) current anticoagulant therapy; or (9) age ≥ 75 years because of the increased risk associated with invasive biopsy procedures.

To exclude advanced dementia, all participants underwent standardized cognitive assessments, including the Korean version of the Mini-Mental State Examination, the Clinical Dementia Rating scale, the Global Deterioration Scale, and the Seoul Neuropsychological Screening Battery-II (Ryu and Yang, 2023).

### Dopamine transporter positron emission tomography/computed tomography

To exclude participants without presynaptic dopaminergic deficits, the ^18^F-FP-CIT positron emission tomography/computed tomography (PET/CT) was performed using a Discovery STE scanner (GE Healthcare, Milwaukee, WI, United States), as previously described (Ryu et al., 2024). Following intravenous administration of ^18^F-FP-CIT (mean dose 3.7 MBq/kg), brain CT images were acquired for attenuation correction, followed by PET imaging approximately 3 h post-injection. PET data were acquired over 10 min.

Mean standardized uptake value ratios (SUVRs) were calculated by an experienced nuclear medicine physician as the ratio of regional tracer uptake to cerebellar uptake. Regions of interest included the bilateral caudate nucleus (anterior and posterior), putamen (anterior, posterior, and ventral), ventral striatum, and overall striatum. For bilateral regions, an asymmetry index was calculated as (SUVR_highest - SUVR_lowest)/(SUVR_highest + SUVR_lowest) × 100, with higher values indicating greater interhemispheric asymmetry.

### SMG biopsy and blood sampling

**FIGURE 2:**
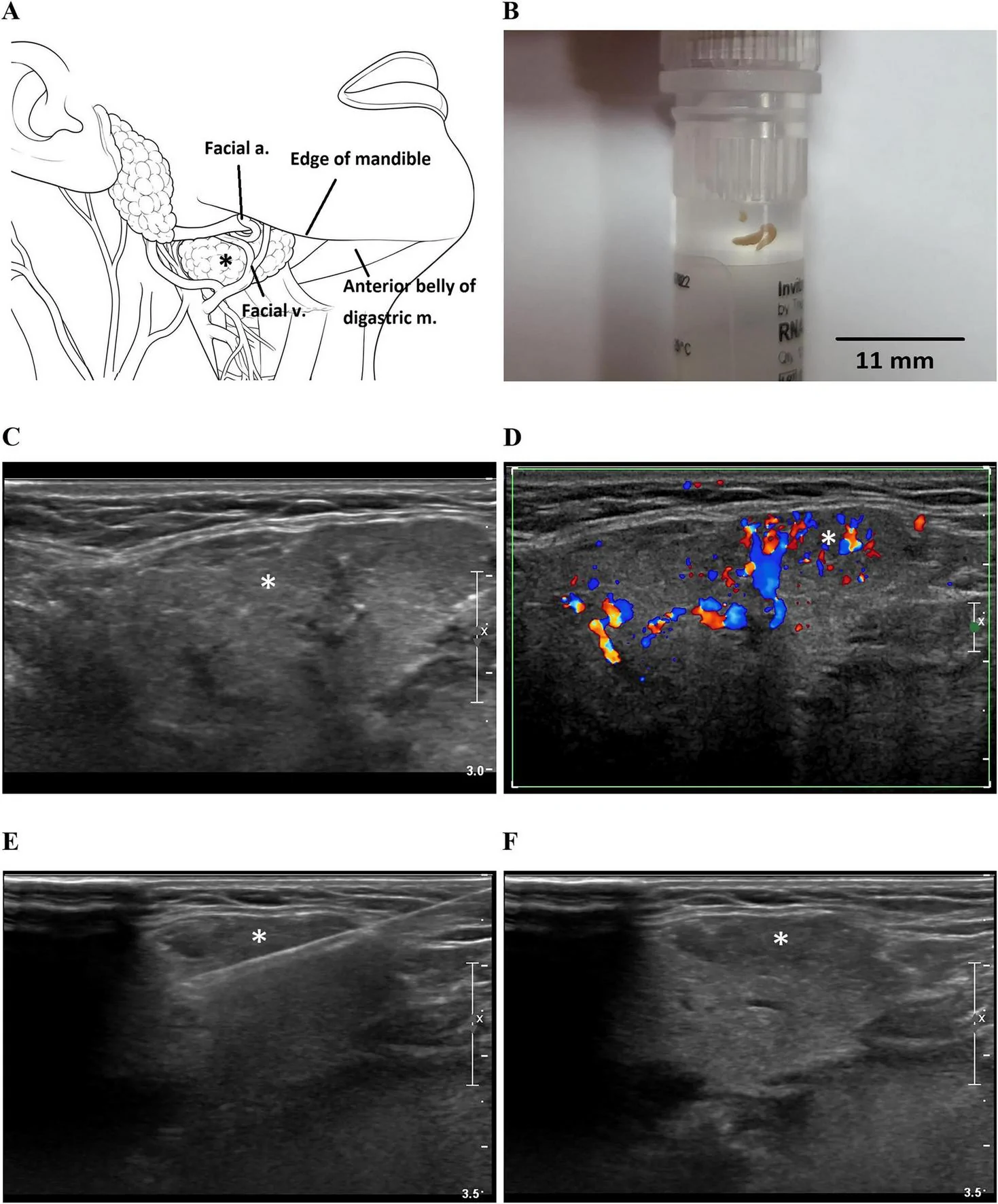
Ultrasound-guided core needle biopsy of the submandibular gland and representative biopsy specimen. **(A)** Schematic illustration of the submandibular gland (SMG) within the submandibular triangle. The submandibular triangle is bounded by the inferior border of the mandible and the anterior and posterior bellies of the digastric muscle. **(B)** Representative SMG biopsy specimen obtained using an 18-gauge core needle biopsy device. Scale bar = 11 mm. **(C–F)** Ultrasound-guided core needle biopsy procedure. **(C)** Gray-scale ultrasound image showing the SMG before biopsy. Examinations were performed using a Philips ultrasound system equipped with a 5–12 MHz linear-array transducer (L12-5). **(D)** Color Doppler ultrasound demonstrating intraglandular vascular structures to facilitate planning of a safe biopsy trajectory. **(E)** Real-time ultrasound-guided insertion of an 18-gauge core needle into the SMG. **(F)** Gray-scale ultrasound image obtained immediately after biopsy, demonstrating no sonographic evidence of significant hemorrhage or structural disruption of the gland. The asterisk indicates the SMG in **(A,C–F)**. Illustration created by the authors based on anatomical landmarks.

In PD patients, SMG biopsy was performed percutaneously under ultrasound guidance on the side with the greater reduction in dopamine transporter (DAT) activity on the ^18^F-FP-CIT PET/CT (Figure 2). Ultrasound examination was performed using a Philips ultrasound system equipped with a 5–12 MHz linear-array transducer (L12-5). Local analgesia was induced with 1% lidocaine prior to tissue retrieval. A disposable 18-gauge biopsy system (Bard Mission Disposable Core Biopsy Instrument; Becton, Dickinson and Company, Franklin Lakes, NJ, United States) was used to collect glandular tissue. Ultrasonographic monitoring was maintained throughout the procedure to immediately detect procedure-related abnormalities, such as active bleeding or focal hematoma formation. Following tissue collection, manual pressure was applied to the biopsy site for approximately 20–30 min to prevent from bleeding. Reference SMG tissue was obtained from surgically excised salivary glands collected during oncologic head and neck procedures, including modified radical neck dissection. Percutaneous biopsy produced tissue cores measuring approximately 1.2 mm in diameter and 7–10 mm in length, whereas surgically excised samples were approximately 3 mm^3^ in size. All tissue specimens were immediately transferred into RNAlater stabilization medium (Thermo Fisher Scientific, Waltham, MA, United States) after removal to prevent RNA degradation. The time from tissue collection to RNAlater immersion was < 1 min in PD patients and 1–20 min in controls.

Overnight fasting venous blood was collected in the morning on the same day as tissue acquisition. Fasting was defined as no caloric consumption for at least 8 h prior to sampling. For RNA analyses, two PAXgene Blood RNA Tubes (BD biosciences, United States), each containing approximately 2.5–3 mL of whole blood, were obtained from each participant; one tube was designated for transcriptome sequencing and the other for small RNA sequencing.

After sample collection, three samples were obtained from each participant: one SMG biopsy specimen preserved in RNAlater solution tube and two peripheral blood samples collected in PAXgene Blood RNA tubes. All samples were stored at 4 °C until RNA extraction was initiated in the laboratory the following day, except for one patient sample that was maintained at 4 °C over the weekend due to Friday sampling.

### RNA extraction

**FIGURE 3:**
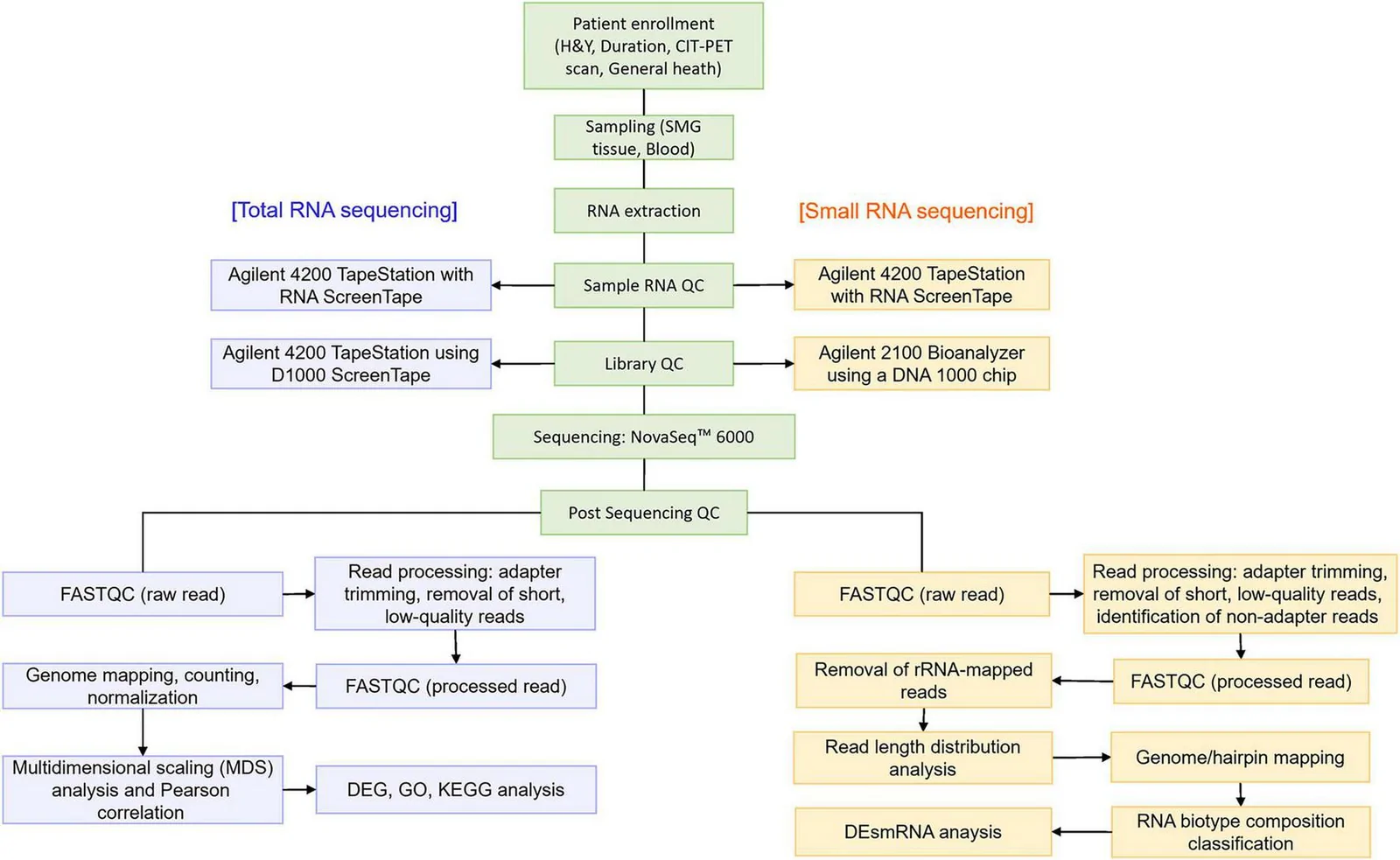
Workflow for technical validation of total RNA-seq and small RNA-seq. Flow chart shows the key stages of sample RNA QC, library QC, and post sequencing QC process.

RNA extraction was performed using protocols optimized for each sample type, as summarized in Table 2. Submandibular gland (SMG) tissue was homogenized in QIAzol Lysis Reagent (Qiagen; Cat. No. 79306) and processed using a TRIzol-based RNA isolation workflow, whereas whole-blood RNA was isolated using the PAXgene Blood RNA Kit (BD Biosciences, Franklin Lakes, NJ, United States) according to the manufacturer’s instructions. A step-by-step protocol for RNA extraction from SMG tissue is provided in Supplementary Table 2. Following RNA extraction, RNA from each SMG specimen was aliquoted into two portions for total RNA sequencing and small RNA sequencing, respectively. All extracted RNA samples underwent RNA integrity assessment prior to cDNA library preparation. RNA integrity was evaluated using the Agilent 4,200 TapeStation System with RNA ScreenTape (Agilent Technologies, Santa Clara, CA, United States), and RNA quantity and integrity were assessed before library preparation. An RINe value of ≥ 7 was used as a recommended quality criterion rather than an absolute exclusion threshold; samples with lower RINe values were retained when library construction was technically feasible. For library preparation, 0.5 and 3 μg of total RNA per sample were used for total RNA-seq and small RNA-seq, respectively, and these input amounts were applied uniformly across all PD and control samples (Figure 3).

**TABLE 2:** Materials and equipment.

Sample type	Assay type	RNA extraction method or kit	cDNA library prep kit	Sequencing platform	Read length (bp)	Library layout
SMG	Total RNA-Seq	RNAlater Tissue Collection followed by Trizol tissue RNA extraction protocol	TruSeq stranded total RNA library prep gold kit	NovaSeq 6000	101	Paired
	Cat No.	Invitrogen (AM7022)	Illumina (20020599)			
	Small RNA-Seq	RNAlater tissue collection followed by Trizol tissue RNA extraction protocol	TruSeq small RNA Library prep kit	NovaSeq 6000	51	Single
	Cat No.	Invitrogen (AM7022)	Illumina (RS-200–0012, 0024, 0036, 0048)			
Blood	Total RNA-Seq	PAXgene blood RNA kit and protocol	TruSeq stranded total RNA library prep globin kit	NovaSeq 6000	101	Paired
	Cat No.	BD bioscience (762165)	Illumina (20020613)			
	Small RNA-Seq	PAXgene blood RNA kit and protocol	TruSeq small RNA library prep kit	NovaSeq 6000	51	Single
	Cat No.	BD bioscience (762165)	Illumina (RS-200–0012, 0024, 0036, 0048)			

SMG, Submandibular Gland Tissue.

### Total RNA library preparation and sequencing

cDNA libraries were prepared using protocols optimized for each sample type and sequencing assay, as summarized in Table 2. For total RNA sequencing, SMG tissue libraries were prepared using the TruSeq Stranded Total RNA Library Prep Gold kit (Illumina; Cat. No. 20020599), whereas blood libraries were prepared using the TruSeq Stranded Total RNA Library Prep Globin kit (Illumina; Cat. No. 20020613). Ribosomal RNA (rRNA) was depleted during library preparation in both workflows, whereas globin RNA was additionally depleted from blood-derived samples. Library quality, including size distribution and concentration, was evaluated for all samples using the Agilent 4,200 TapeStation with D1000 ScreenTape (Agilent Technologies, Santa Clara, CA, United States) (Figure 3). Libraries that passed quality control were pooled and sequenced on an Illumina NovaSeq 6000 platform (Illumina, San Diego, CA, United States). Total RNA libraries were sequenced using paired-end 2 × 101 bp reads.

### Small RNA library preparation and sequencing

For small RNA sequencing, libraries from both SMG tissue and peripheral blood were prepared using the TruSeq Small RNA Library Prep Kit (Illumina) (Table 2). Library quality, including size distribution and concentration, was evaluated for all samples using the Agilent 2,100 Bioanalyzer with the DNA 1000 chip (Agilent Technologies, Santa Clara, CA, United States) (Figure 3). Libraries that passed quality control were pooled and sequenced on an Illumina NovaSeq 6000 platform (Illumina, San Diego, CA, United States). Small RNA libraries were sequenced using single-end 51 bp reads.

### Post-sequencing quality control

Sequencing base quality of the raw and processed FastQ files were assessed using FastQC.^1^ For both total RNA-seq and small RNA-seq, per-base sequence quality (Phred scores), GC content were inspected to evaluate sequencing performance before and after read processing. Phred quality scores represent the probability of an incorrect base call; for example, a Phred score of 30 corresponds to an estimated error rate of 1 in 1,000. For total RNA-seq, principal component analysis (PCA) based on rlog-transformed gene expression counts and Pearson’s correlation analysis were performed to assess sample-level consistency following data processing. For small RNA-seq, read length distribution was additionally evaluated after read processing to confirm enrichment of mature small RNAs and the effective removal of short degradation products and sequencing artifacts.

### Total RNA bioinformatic analysis

Adapter sequences introduced during library preparation were removed using Trimmomatic (v0.38)^2^ with the Illumina TruSeq3 paired-end adapter reference, followed by quality trimming and filtering. The resulting trimmed reads were aligned to the human reference genome (GRCh38/hg38) using HISAT2.^3^ Gene and transcript abundances were estimated using StringTie (v2.1.3b).^4^ Gene-level read counts generated by StringTie were normalized using the relative log expression (RLE) method implemented in DESeq2.^5^ Differential expression analysis was performed separately for SMG and blood samples using the negative binomial Wald test implemented in DESeq2.

### Small RNA bioinformatic analysis

The 3’ Illumina TruSeq small RNA adapter sequence (TGGAATTCTCGGGTGCCAAGG) was removed using Cutadapt (v2.8).^6^ Adapter-trimmed reads were subsequently quality-filtered, and reads shorter than 17 nt or containing ambiguous nucleotides (“N”) were discarded. To minimize interference from abundant ribosomal RNA-derived fragments, the remaining reads were aligned to human 45S pre-rRNA and mitochondrial rRNA reference sequences, and rRNA-mapped reads were removed prior to downstream analyses. Processed reads were collapsed into unique sequences defined by identical sequence and length before alignment. Known miRNAs were identified by alignment to miRBase release 22.1,^7^ whereas other small RNA classes, including piRNAs, tRNAs, snRNAs, and snoRNAs, were annotated using RNAcentral release 14.0.^8^ Novel miRNAs were predicted using miRDeep2^9^ based on read distribution across putative precursor loci and RNAfold-derived secondary structures. For broader small RNA annotation, processed reads were aligned to RNAcentral using Bowtie^10^ for short RNAs (≤ 50 nt) and Bowtie2 for longer structured RNAs (≥ 50 nt). Reads that could not be assigned to curated RNA classes were subsequently aligned to the human reference genome and repeat sequences. RNA biotype composition was calculated from uniquely mapped reads using a hierarchical annotation strategy to avoid multiple assignment. Mature small RNAs with zero counts in more than 51% of samples were excluded from downstream analyses, and the remaining counts were normalized using the trimmed mean of M-values (TMM) method implemented in edgeR. Differential expression analysis was performed using the exact test in edgeR, as described previously (Choi et al., 2025).

## Results

### Participants enrollment

A total of 13 participants were enrolled in the study, comprising seven patients with *de novo* Parkinson’s disease (PD) and six age- and sex-matched, neurologically unaffected surgical controls (Table 1). All PD patients were *de novo* and drug-naïve at enrollment. The mean age of the PD and control groups was 64.1 ± 6.2 and 61.7 ± 6.2 years, respectively, with comparable sex distributions (4 males and 3 females in the PD group; 3 males and 3 females in the control group). At the time of sampling, PD disease duration ranged from 6 to 48 months. Disease severity varied across participants, with Hoehn and Yahr stages ranging from 1 to 3 and UPDRS Part III scores ranging from 3 to 52 (Table 1).

### Sample RNA quality control

**FIGURE 4:**
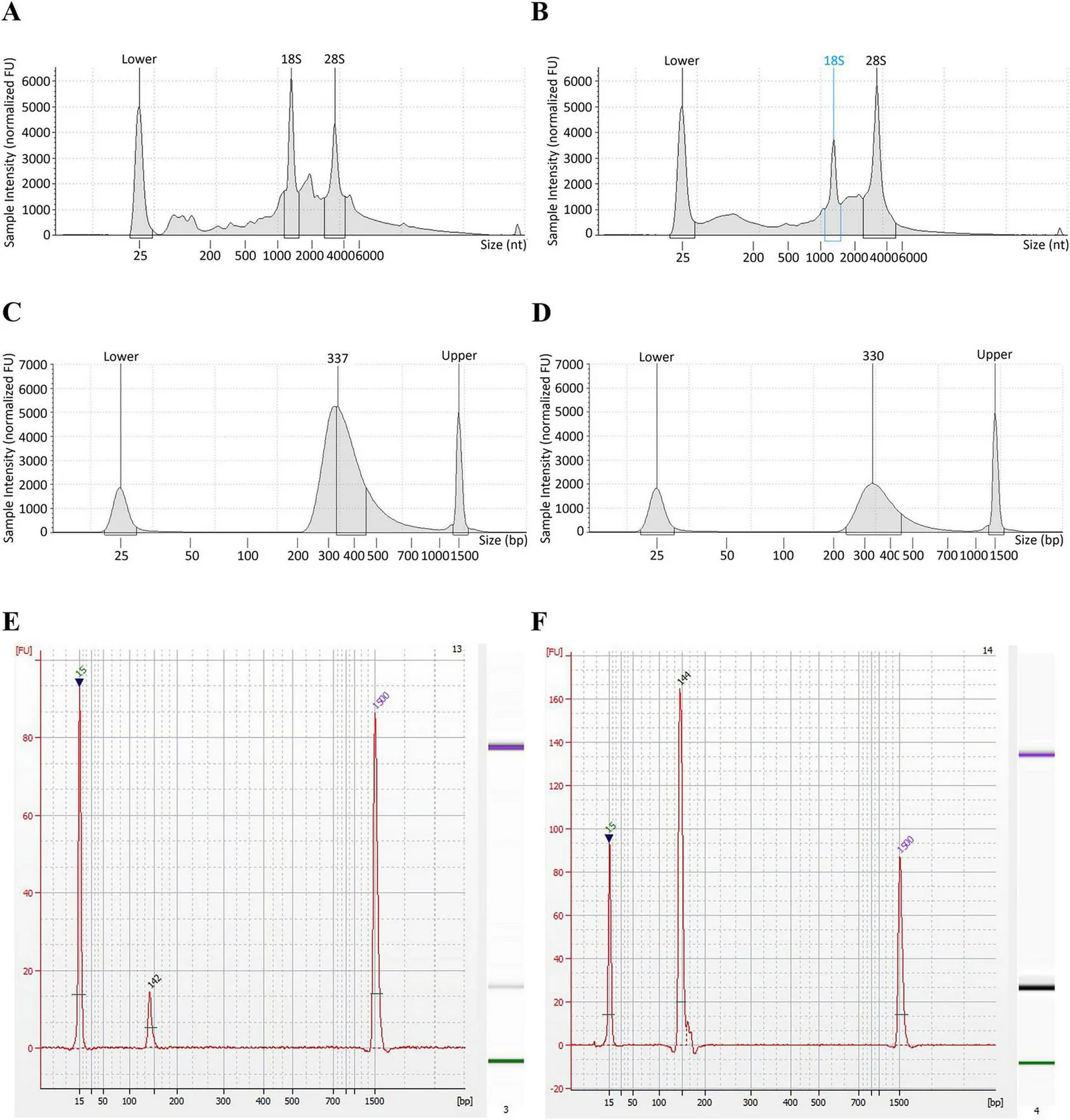
Representative images of pre-sequencing RNA integrity and cDNA library quality control. **(A,B)** RNA integrity assessment. **(A)** SMG tissue RNA (RINe = 8.5). **(B)** Blood RNA (RINe = 8.1). **(C,D)** Total RNA-seq cDNA library quality. **(C)** SMG tissue library. **(D)** Blood library. **(E,F)** Small RNA-seq cDNA library quality. **(E)** SMG tissue library. **(F)** Blood library. RINe values indicates high RIN integrity, and the peak fragment size reflects the optimal insert size of each cDNA library. All samples shown are from PD_donor7.

RINe values were ≥ 6 for all samples except for one blood specimen with a RINe value of 4.9 (mean RINe = 7.88, range 4.9–8.8; Table 3). All samples were included in the downstream analyses. Representative electropherograms (Figures 4A,B) showed well-defined 18S and 28S ribosomal RNA peaks with minimal low-molecular-weight degradation products, consistent with the overall high RNA integrity of the study samples. The single blood sample with a RINe value of 4.9 was collected on a Friday and underwent RNA extraction after storage at 4°C over the weekend, whereas all other samples were processed on the following day. The prolonged storage period may have contributed to the lower RNA integrity observed in this sample. Despite the small biopsy size (approximately 1.2 × 7–10 mm), RNA yields from all SMG specimens were sufficient to generate both total RNA and small RNA sequencing libraries (Table 3).

**TABLE 3:** Sample RNA QC and library QC metrics.

	Sample RNA QC						Library QC		
Patient ID	Sample type	Conc. (ng/μL)	Final RNA vol. (μL)	Total RNA yield μg	RINe	rRNA ratio (28S/18S)	Library Conc. (ng/μL)	Library Conc. (nM)	Library size (bp)
PD_donor1	Total-T	159.895	30	4.797	8	1.2	26.2	116	327
	Total-B	27.156	50	1.358	7	1.1	6.9	29.3	345
	smR-T	159.895	30	4.797	8	1.2	10.54	115.05	141
	smR-B	108.624	50	5.431	7	1.2	33.88	362.01	144
PD_donor2	Total-T	67.17	12	0.806	8.6	1	26.1	112	336
	Total-B	8.33	75	0.625	8.3	3.3	26.5	115	325
	smR-T	67.17	45	3.023	8.6	1	8.94	98.24	140
	smR-B	47.982	55	2.639	7.3	1.8	23.52	251.28	144
PD_donor3	Total-T	212.565	20	4.251	7.6	1	27.7	113	354
	Total-B	38.831	15	0.582	8.2	2	0.6	3.64	328
	smR-T	212.565	30	6.377	7.6	1.1	12.75	138.1	142
	smR-B	38.831	55	2.136	8.2	2	45.25	486.84	143
PD_donor4	Total-T	194.261	20	3.885	7.9	1	31.5	130	351
	Total-B	77.031	20	1.541	7.7	1.2	17.4	72.2	355
	smR-T	194.261	30	5.828	8	1	11.4	123.46	142
	smR-B	77.031	50	3.8527	7.7	1.2	36.73	389.74	145
PD_donor5	Total-T	175.302	10	1.753	8.3	1.4	33.8	138	349
	Total-B	41.054	15	0.616	6.3	1.7	47.9	205	337
	smR-T	175.302	45	7.889	8.3	1.4	6.5	70.93	141
	smR-B	69.857	45	3.144	4.9	0.7	24.93	266.34	144
PD_donor6	Total-T	146.063	20	2.921	8.6	1.2	14.8	60.5	350
	Total-B	262.663	20	5.253	8.4	1.5	4.18	16.4	380
	smR-T	146.063	50	7.303	8.6	1.2	3.9	42.29	142
	smR-B	262.663	30	7.88	8.4	1.5	13.5	147.32	141
PD_donor7	Total-T	140.04	25	3.501	8.5	1.1	19.1	82.6	330
	Total-B	143.68	25	3.592	8.1	2.3	48.2	206	337
	smR-T	140.04	25	3.501	8.5	1.1	3.27	35.47	142
	smR-B	143.68	25	3.592	8.1	2.3	34.67	370.42	144
CTL_donor1	Total-T	393.252	50	19.663	7.8	1	7.65	32.3	344
	Total-B	22.219	50	1.111	7.7	2.5	16.9	70.5	347
	smR-T	393.252	50	19.663	7.8	1	12.36	132.93	143
	smR-B	61.82	50	3.091	6	1.5	27.74	298.47	143
CTL_donor2	Total-T	496.129	20	9.923	8.8	1	37.8	156	350
	Total-B	84.121	20	1.682	8.6	1.5	18.8	76.3	352
	smR-T	496.129	40	19.845	8.8	1	11.72	126.95	142
	smR-B	84.121	50	4.206	8.6	1.5	30.51	323.71	145
CTL_donor3	Total-T	494.478	10	4.945	8	1.1	22.6	91.5	355
	Total-B	68.493	15	1.027	7.3	1.2	7.64	32	347
	smR-T	494.478	40	19.779	8	1.1	5.44	59.73	140
	smR-B	68.493	45	3.082	7.3	1.2	25.1	271.95	142
CTL_donor4	Total-T	340.733	50	17.037	8.5	1	42.5	172	354
	Total-B	202.951	50	10.148	8.6	3.5	53.3	226	340
	smR-T	340.733	50	17.037	8.5	1	7	76.94	140
	smR-B	202.951	50	10.148	8.6	3.5	34.16	364.98	144
CTL_donor5	Total-T	317.788	20	6.356	7.4	1	30.1	129	336
	Total-B	249.836	20	4.997	6.2	1	95.4	407	334
	smR-T	317.788	80	25.423	7.4	1	17.39	188.54	142
	smR-B	249.836	35	8.744	6.2	1	8.11	89.17	140
CTL_donor6	Total-T	1100.718	25	27.518	8.2	1.5	35.1	152	327
	Total-B	200.926	25	5.023	8.4	3.2	8.41	36.6	338
	smR-T	1100.718	25	27.518	8.2	1.5	19.88	213.86	143
	smR-B	200.926	25	5.023	8.4	3.2	5.82	63.03	142

### cDNA library quality control

For total RNA sequencing, the cDNA libraries of all samples showed a peak fragment size of approximately 300–400 bp, consistent with optimal insert sizes for paired-end sequencing. For small RNA sequencing, the expected library size showed a predominant peak at approximately 140–150 bp, corresponding to adapter-ligated small RNA fragments (Table 3). Representative electropherograms demonstrated cDNA library quality for total RNA sequencing (Figures 4C,D), and small RNA sequencing (Figures 4E,F).

### Post-sequencing quality assessment

**FIGURE 5:**
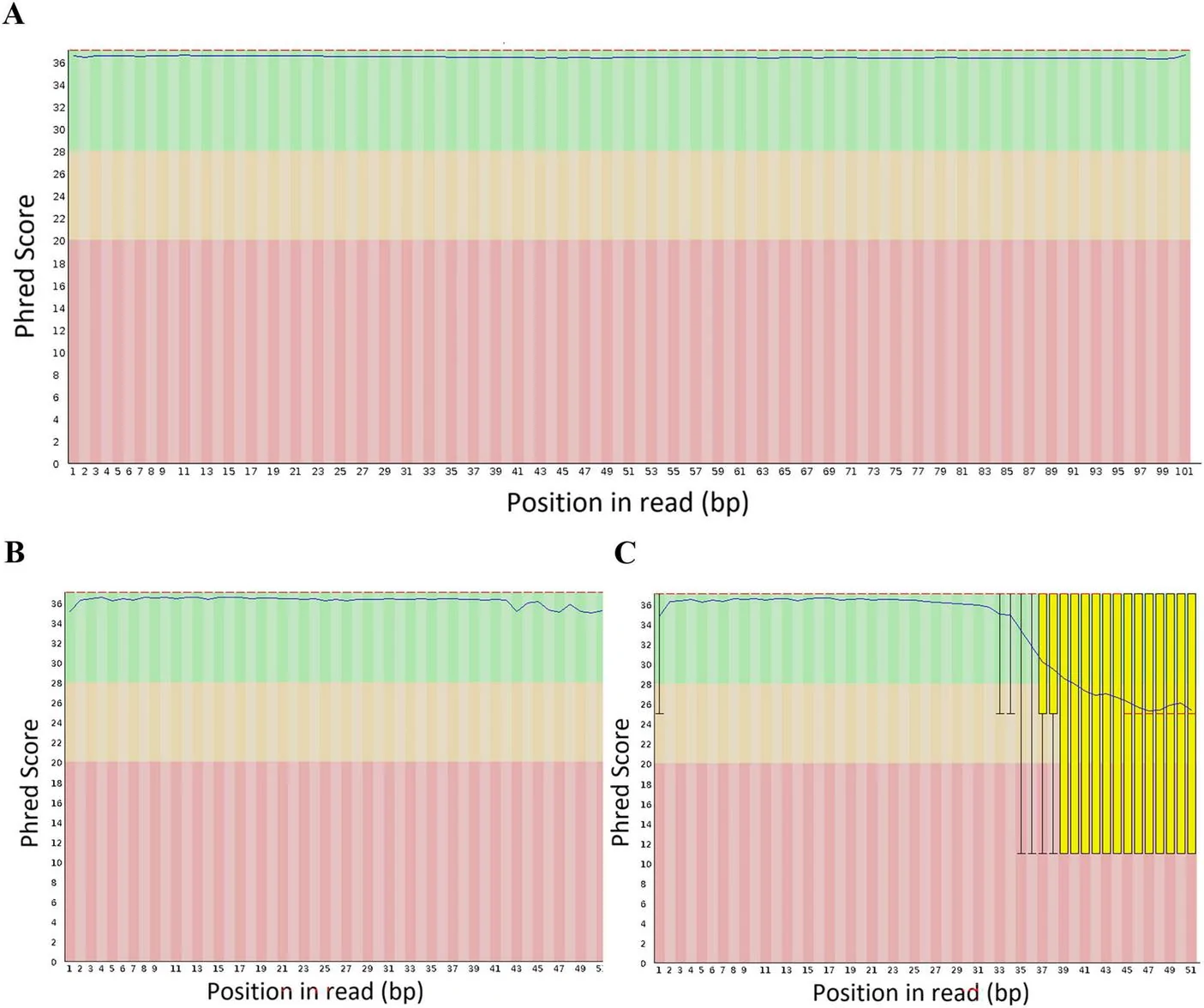
Per-base sequence quality for total RNA-seq and small RNA-seq. Example of FastQC plots (Phred scores) from PD_donor7 are shown. The blue line indicates the mean Phred score at each position, and boxplots show the score distribution (green, high quality; orange, moderate; red, low). **(A)** Trimmed total RNA-seq reads show consistently high base quality across read positions (mean Phred > 30). **(B)** Raw small RNA-seq reads also exhibit high per-base quality across the full read length, indicating robust sequencing performance. **(C)** After adapter trimming and quality filtering, small RNA-seq reads retain high quality across positions corresponding to the predominant small RNA insert lengths (< 35 bp). The decline in quality beyond ∼35 bp is confined to a small fraction of longer reads and low read coverage at late-cycle positions, and therefore does not affect the main small RNA population, consistent with the read length distribution peak at 18–35 bp (Figure 6).

**FIGURE 6:**
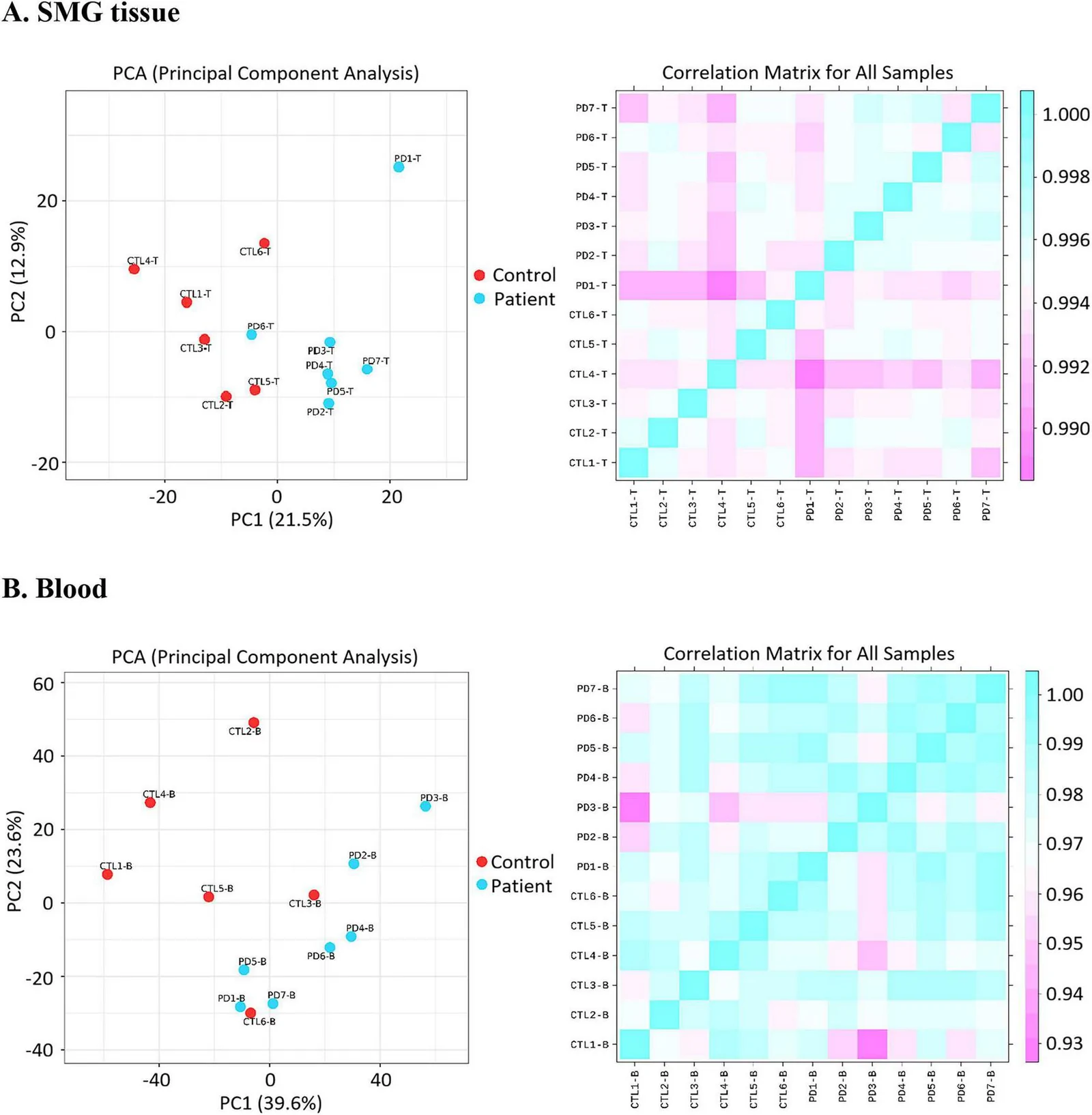
PCA plot and correlation heatmap for total RNA-seq. **(A)** Submandibular gland (SMG) samples. **(B)** Blood samples. PCA shows that samples from the same group (control vs. patient) are relatively well clustered together. The Pearson correlation analysis and the pairwise heatmap plot show high correlations within each group, with correlation coefficients of > 0.99 for SMG **(A)** and > 0.93 for blood **(B)**.

For total RNA-seq, adapter trimming and quality filtering generated high-quality reads suitable for downstream analyses (Table 4). Across all libraries, Q30 scores ranged from 94.88 to 96.63% for SMG samples and from 94.96 to 96.44% for blood samples, indicating consistently high sequencing quality (Figure 5A). PCA of rlog-transformed gene expression counts demonstrated clear separation between PD and control samples. The first two principal components explained 21.5 and 12.9% of the total variance in SMG samples and 39.6 and 23.6% in blood samples, respectively. Sample-to-sample similarity was consistently high, with Pearson’s correlation coefficients ranging from 0.99 to 1.00 for SMG samples and from 0.93 to 1.00 for blood samples (Figure 6).

**TABLE 4:** Post-sequencing quality control for total RNA-seq.

Sample ID	Sample type	SRA run accession No.	Raw total bases (bp)	Raw reads count	Trimmed total bases (bp)	Trimmed reads count	GC (%)	Q30 (%)	Mapped reads (%)	Unmapped read (%)
PD_donor1	SMG	SRR26372877	12,104,247,838	119,844,038	11,789,980,341	117,441,252	49.57	96.26	115,537,783 (98.38%)	1,903,469 (1.62%)
	Blood	SRR26372857	13,022,164,926	128,932,326	12,573,528,619	125,293,514	49.03	96.28	122,675,964 (97.91%)	2,617,550 (2.09%)
PD_donor2	SMG	SRR26372876	11,669,301,842	115,537,642	11,296,468,363	112,437,100	48.18	95.39	110,845,586 (98.58%)	1,591,514 (1.42%)
	Blood	SRR26372856	12,548,733,082	124,244,882	12,133,624,063	120,885,178	44.14	94.88	118,891,146 (98.35%)	1,994,032 (1.65%)
PD_donor3	SMG	SRR26372845	10,425,832,060	103,226,060	10,173,481,347	101,212,842	47.96	96.11	100,001,211 (98.8%)	1,211,631 (1.2%)
	Blood	SRR26372854	11,983,347,808	118,647,008	11,656,324,424	116,356,444	46.81	96.16	109,785,217 (94.35%)	6,571,227 (5.65%)
PD_donor4	SMG	SRR26372834	12,409,251,476	122,863,876	12,113,914,413	120,535,490	48.67	96.05	119,087,587 (98.8%)	1,447,903 (1.2%)
	Blood	SRR26372875	12,115,904,046	119,959,446	11,805,263,792	117,487,294	47.42	95.95	116,064,151 (98.79%)	1,423,143 (1.21%)
PD_donor5	SMG	SRR26372872	12,615,272,084	124,903,684	12,321,070,247	122,538,002	47.65	96.63	121,112,059 (98.84%)	1,425,943 (1.16%)
	Blood	SRR26372855	13,247,751,860	131,165,860	12,921,033,168	128,580,558	48.99	96.6	126,942,917 (98.73%)	1,637,641 (1.27%)
PD_donor6	SMG	SRR26372861	12,364,892,680	122,424,680	12,056,601,042	119,957,636	46.06	96.34	118,066,072 (98.42%)	1,891,564 (1.58%)
	Blood	SRR26372853	13,352,627,634	132,204,234	13,012,104,167	129,520,892	45.78	96.06	127,616,407 (98.53%)	1,904,485 (1.47%)
PD_donor7	SMG	SRR26372858	13,318,871,818	131,870,018	13,013,821,850	129,480,312	49.71	96.27	127,499,631 (98.47%)	1,980,681 (1.53%)
	Blood	SRR26372852	13,312,651,430	131,808,430	12,928,108,628	128,895,114	45.91	95.04	125,441,777 (97.32%)	3,453,337 (2.68%)
CTL_donor1	SMG	SRR26372851	13,337,317,650	132,052,650	12,973,708,618	129,218,890	47.53	94.96	127,431,634 (98.62%)	1,787,256 (1.38%)
	Blood	SRR26372844	13,327,126,548	131,951,748	12,887,761,811	128,541,932	48.79	95.77	126,139,760 (98.13%)	2,402,172 (1.87%)
CTL_donor2	SMG	SRR26372850	11,277,221,862	111,655,662	11,005,173,693	109,517,692	47.18	95.92	108,052,810 (98.66%)	1,464,882 (1.34%)
	Blood	SRR26372843	11,414,023,130	113,010,130	11,113,211,221	110,567,262	49.5	96.14	109,196,123 (98.76%)	1,371,139 (1.24%)
CTL_donor3	SMG	SRR26372849	11,736,728,634	116,205,234	11,458,469,245	114,002,876	47.45	96.19	112,590,436 (98.76%)	1,412,440 (1.24%)
	Blood	SRR26372842	10,044,507,166	99,450,566	9,812,841,862	97,602,562	51.73	96.34	96,174,173 (98.54%)	1,428,389 (1.46%)
CTL_donor4	SMG	SRR26372848	13,339,358,456	132,072,856	13,021,825,707	129,580,464	46.49	96.27	127,320,945 (98.26%)	2,259,519 (1.74%)
	Blood	SRR26372841	13,220,454,388	130,895,588	12,867,900,659	128,144,894	46.8	96.1	125,989,910 (98.32%)	2,154,984 (1.68%)
CTL_donor5	SMG	SRR26372847	13,037,128,076	129,080,476	12,751,059,887	127,023,040	47.98	96.07	124,861,005 (98.3%)	2,162,035 (1.7%)
	Blood	SRR26372840	13,326,098,166	131,941,566	12,964,338,463	129,601,164	46.58	95.55	126,816,955 (97.85%)	2,784,209 (2.15%)
CTL_donor6	SMG	SRR26372846	10,883,616,580	107,758,580	10,696,673,131	106,418,902	48.67	96.44	104,997,475 (98.66%)	1,421,427 (1.34%)
	Blood	SRR26372839	11,843,171,928	117,259,128	11,585,650,615	115,436,508	48.68	96.44	113,483,211 (98.31%)	1,953,297 (1.69%)

**FIGURE 7:**
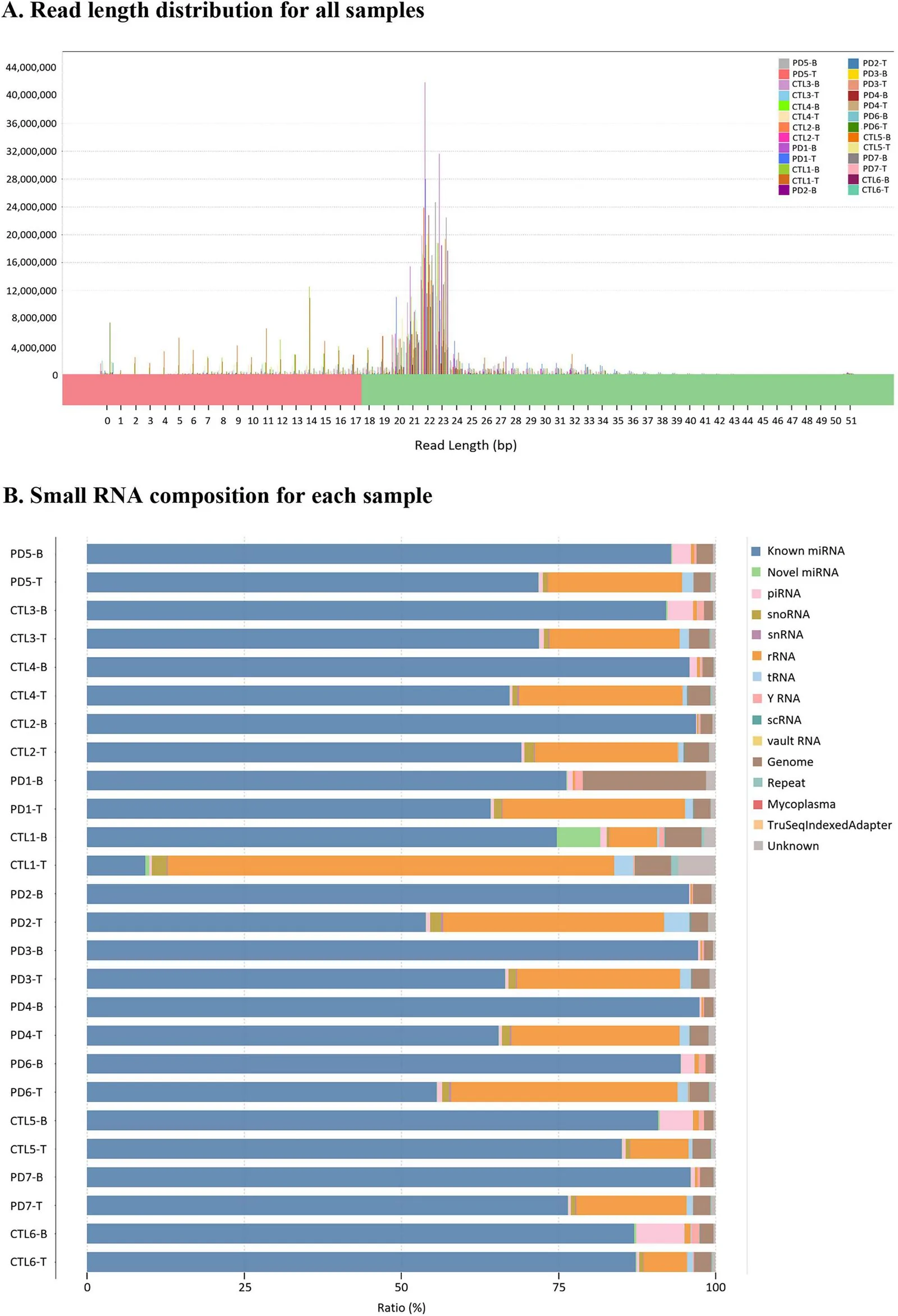
Read length distribution and sequence composition in small RNA-seq. **(A)** Read length distribution for all samples, showing a predominant peak at ∼22–23 nt consistent with miRNA-sized reads, with additional peaks/shoulders reflecting other small RNA species. **(B)** Small RNA composition for each sample, including known/novel miRNAs as well as other small RNAs.

For small RNA-seq, raw sequencing reads exhibited consistently high per-base quality scores, indicating robust sequencing performance (Figure 5B and Table 5). Because small RNA libraries contain short inserts, the 3’ portions of raw reads predominantly corresponded to adapter sequences despite the high sequencing quality. Following adapter trimming and quality filtering, per-base quality remained above a Phred score of 30 across most read positions, with a slight decline beyond approximately 35 bp observed only in a minor subset of longer reads (Figure 5C). Read length distribution showed a pronounced peak at 21–23 nt, corresponding to mature miRNAs, together with a smaller population of reads between 24 and 30 nt, consistent with other classes of small RNAs such as piRNAs. Reads shorter than 17 nt were infrequent, indicating effective removal of degraded RNA fragments and sequencing artifacts (Figure 7A and Supplementary Figure 1).

**TABLE 5:** Post-sequencing quality control for small RNA-seq.

Sample ID	Sample type	SRA run accession no.	Raw total bases (bp)	Raw reads count	Trimmed total bases (bp)	Trimmed reads count	GC (%)	Q30 (%)	Non-adaptor read count	GC (%)	Q30 (%)	Short read count (%)	Low quality read count (%)
PD_donor1	SMG	SRR26372838	4,615,194,612	90,494,012	1,948,856,907	82,889,109	48.44	96.2	250,545	43.77	63.23	7,296,053 (8.06%)	58,305 (0.06%)
	Blood	SRR26372830	4,885,478,751	95,793,701	2,129,967,482	95,148,220	63.16	93.4	270,839	51.61	60.36	340,814 (0.36%)	33,828 (0.04%)
PD_donor2	SMG	SRR26372837	1,775,467,590	34,813,090	818,632,285	32,814,221	50.04	95.04	139,283	46.33	59.65	1,838,887 (5.28%)	20,699 (0.06%)
	Blood	SRR26372829	1,675,028,955	32,843,705	740,301,573	32,720,555	61.2	95.97	65,344	46.65	60.94	36,534 (0.11%)	21,272 (0.06%)
PD_donor3	SMG	SRR26372836	2,035,777,659	39,917,209	875,008,274	37,529,574	48	95.11	168,219	48.85	55.63	2,198,920 (5.51%)	20,496 (0.05%)
	Blood	SRR26372828	2,067,805,047	40,545,197	894,605,043	40,390,864	61.39	96.38	71,403	49.11	57.23	61,085 (0.15%)	21,845 (0.05%)
PD_donor4	SMG	SRR26372835	2,418,291,480	47,417,480	1,057,591,877	44,947,577	48.14	95.7	207,894	48.88	56.21	2,237,459 (4.72%)	24,550 (0.05%)
	Blood	SRR26372827	2,374,987,227	46,568,377	1,026,848,357	46,417,005	61.42	96.35	63,075	49.71	59	62,998 (0.14%)	25,299 (0.05%)
PD_donor5	SMG	SRR26372833	2,295,423,912	45,008,312	860,311,251	37,968,507	47.24	96.62	74,498	42.54	54.05	6,956,536 (15.46%)	8,771 (0.02%)
	Blood	SRR26372826	2,494,229,919	48,906,469	1,072,331,223	47,344,045	60.41	97.25	39,338	48.49	57.69	1,513,735 (3.1%)	9,351 (0.02%)
PD_donor6	SMG	SRR26372832	2,393,441,832	46,930,232	758,016,751	32,262,806	50.1	95.56	81,214	49.07	63.11	14,578,081 (31.06%)	8,131 (0.02%)
	Blood	SRR26372874	1,942,549,863	38,089,213	836,883,767	37,547,545	55.12	97.06	39,472	45.81	62.4	494,959 (1.3%)	7,237 (0.02%)
PD_donor7	SMG	SRR26372831	1,896,846,978	37,193,078	687,238,710	30,657,544	46.4	96.32	58,087	40.48	53.89	6,470,882 (17.4%)	6,565 (0.02%)
	Blood	SRR26372873	2,476,876,863	48,566,213	1,070,349,578	47,709,501	61.11	96.98	36,481	49.21	58.47	811,012 (1.67%)	9,219 (0.02%)
CTL_donor1	SMG	SRR26372871	4,986,440,952	97,773,352	862,588,833	36,504,495	53.55	97.16	186,464	47.27	56.54	61,055,662 (62.45%)	26,731 (0.03%)
	Blood	SRR26372865	4,976,939,499	97,587,049	1,126,653,236	51,476,848	56.44	95.51	130,686	39.01	55.67	45,952,109 (47.09%)	27,406 (0.03%)
CTL_donor2	SMG	SRR26372870	2,491,906,767	48,860,917	1,060,090,975	45,910,359	47.49	95.06	171,829	47.56	55.61	2,754,100 (5.64%)	24,629 (0.05%)
	Blood	SRR26372864	2,512,292,997	49,260,647	1,098,332,505	49,071,567	61.09	96.75	89,962	50.47	57.85	72,200 (0.15%)	26,918 (0.05%)
CTL_donor3	SMG	SRR26372869	2,228,845,707	43,702,857	783,405,346	34,740,038	47.52	96.38	57,194	46.15	57.89	8,898,015 (20.36%)	7,610 (0.02%)
	Blood	SRR26372863	2,505,275,346	49,123,046	1,041,771,108	46,840,119	58.34	97.02	61,191	42.91	54.48	2,212,750 (4.5%)	8,986 (0.02%)
CTL_donor4	SMG	SRR26372868	2,348,875,635	46,056,385	637,794,608	28,630,780	47.88	96.09	125,635	48.38	81.66	17,293,699 (37.55%)	6,271 (0.01%)
	Blood	SRR26372862	2,303,148,984	45,159,784	991,997,800	44,306,414	60.28	97.56	40,649	45.28	55.58	804,087 (1.78%)	8,634 (0.02%)
CTL_donor5	SMG	SRR26372867	2,499,992,154	49,019,454	937,530,778	42,581,416	44.76	96.4	48,765	40.44	56.58	6,380,546 (13.02%)	8,727 (0.02%)
	Blood	SRR26372860	2,323,579,125	45,560,375	1,002,429,136	44,337,275	56.42	97.39	68,969	48.45	71.69	1,145,325 (2.51%)	8,806 (0.02%)
CTL_donor6	SMG	SRR26372866	1,847,107,545	36,217,795	680,819,151	30,782,718	44.58	96.41	33,357	41.33	59.31	5,395,432 (14.9%)	6,288 (0.02%)
	Blood	SRR26372859	2,185,051,344	42,844,144	949,757,850	41,568,699	58.17	97.29	38,230	49.98	59.55	1,228,962 (2.87%)	8,253 (0.02%)

### Total RNA mapping and annotation

After alignment to the human reference genome (GRCh38/hg38), 97.32–99.10% of SMG reads and 94.35–98.76% of blood reads were successfully mapped, with only 0.90–2.68% and 1.24–5.65% of reads remaining unmapped, respectively (Table 4). Reference-guided transcript assembly using StringTie identified 37,660 annotated genes and 62,423 putative novel genes across all 26 total RNA sequencing libraries. In total, 230,224 transcripts were assembled, including 135,436 annotated transcripts and 94,788 putative novel transcripts. Gene-level read counts generated from the reference-guided annotation were subsequently used for tissue-specific differential expression analyses of SMG and blood samples.

### Small RNA mapping and annotation

rRNA-derived reads were successfully removed before downstream analyses, and the corresponding filtering statistics are summarized in Figure 7B and Supplementary Table 3. Alignment statistics for the human reference genome (GRCh38) and precursor miRNA hairpin sequences are presented in Supplementary Tables 4, 5, respectively. Hierarchical annotation enabled robust classification of known miRNAs together with multiple classes of small non-coding RNAs, including piRNAs, tRNAs, snRNAs, and snoRNAs, while minimizing multiple assignment of sequencing reads. RNA biotype composition estimated from uniquely mapped reads is summarized in Supplementary Tables 6, 7.

## Discussion

Increasing evidence indicates that pathological protein aggregation is a central driver of neurodegenerative diseases (Hsiao et al., 1996; Recasens et al., 2014; Masuda-Suzukake et al., 2013). Consequently, pathological proteins are increasingly recognized as the primary therapeutic targets for disease-modifying interventions across neurodegenerative disorders (Sidoryk-Węgrzynowicz et al., 2024), including amyloid-β in Alzheimer’s disease (van Dyck et al., 2023; Sims et al., 2023), α-synuclein in Parkinson’s disease (Pagano et al., 2022), and SOD1 (Miller et al., 2026) in amyotrophic lateral sclerosis. Although several protein-targeting therapies have demonstrated clinical benefit (van Dyck et al., 2023; Sims et al., 2023), reliable peripheral biomarkers that reflect disease-associated molecular changes remain limited.

To address this need, recent transcriptomic studies have increasingly focused on accessible peripheral tissues that may capture disease-related molecular alterations. In Alzheimer’s disease, single-cell RNA sequencing of the olfactory mucosa has identified neuronal and non-neuronal transcriptional signatures associated with disease pathology, highlighting the potential of peripheral tissues as a surrogate for central nervous system molecular changes (D’Anniballe et al., 2026). Similarly, in Parkinson’s disease, transcriptomic analyses of skin biopsies have demonstrated the feasibility of peripheral molecular profiling and suggested that RNA sequencing may facilitate mechanistic disease stratification relevant to the development of disease-modifying therapies (Kurvits et al., 2021; Carling et al., 2020). However, such studies remain limited in number and require further validation. Compared with skin, transcriptomic characterization of the submandibular gland (SMG), a peripheral tissue implicated in Parkinson’s disease pathology, remains particularly scarce despite increasing evidence supporting its potential relevance (Choi et al., 2025).

The protocol presented here addresses an important technical challenge in Parkinson’s disease research by providing a validated workflow for transcriptomic profiling of paired submandibular gland (SMG) tissue and peripheral blood obtained from untreated patients. The workflow encompasses participant recruitment, ultrasound-guided SMG biopsy, RNA extraction, library preparation, sequencing, and bioinformatic processing, and consistently produced RNA and sequencing data of sufficient quality despite the small size of the biopsy specimens. Because *in vivo* SMG tissue from untreated patients is difficult to obtain and rarely available for research, this protocol provides a practical framework for generating transcriptomic data from a clinically accessible peripheral tissue. The accompanying dataset demonstrates the feasibility of the workflow and provides paired SMG and blood RNA profiles from patients with *de novo*, drug-naïve PD.

Beyond the present study, this workflow may support future investigations of peripheral molecular alterations in Parkinson’s disease. The paired availability of SMG and peripheral blood transcriptomes allows comparison of tissue-associated and circulating RNA profiles and may help evaluate candidate molecular signatures across peripheral compartments. The protocol may also be adapted in future studies incorporating complementary approaches, such as single-cell RNA sequencing, spatial transcriptomics, or other multi-omics analyses.

Several limitations should be considered when interpreting the data generated using this protocol. First, the sample size was necessarily small because ultrasound-guided SMG biopsy in untreated patients with Parkinson’s disease is an invasive procedure that is rarely performed in clinical research; therefore, larger independent cohorts will be required to validate disease-associated molecular signatures. A further limitation is the lack of histopathological confirmation of α-synuclein pathology and tissue composition in individual SMG biopsy specimens, as histological heterogeneity may influence RNA expression profiles. Future studies should therefore consider allocating a small portion of each biopsy specimen for histopathological and α-synuclein assessment, or adopting analytical platforms that enable simultaneous evaluation of α-synuclein pathology and transcriptomic signals within the same specimen, such as spatial transcriptomics. Another major limitation of this study is that the control SMG specimens were obtained from patients undergoing surgery for head and neck cancer and were therefore not anatomically or procedurally matched to the ultrasound-guided core biopsy specimens obtained from patients with PD. In the control group, laterality was necessarily determined by the side of surgery and could not be matched to the side selected in the PD group. Control tissue was collected from grossly normal-appearing intraglandular SMG parenchyma without gross tumor involvement; however, the exact intraglandular location, distance from the primary tumor, and SMG-specific histopathological status were not systematically documented. In addition, the underlying oncological environment, surgical manipulation, ischemic exposure, and potential differences in cellular composition may have influenced the transcriptomic profiles, even in grossly normal-appearing tissue. We therefore consider these specimens reasonably comparable for assessing the technical feasibility of SMG transcriptomic profiling, while acknowledging that they are not fully equivalent for establishing PD-specific transcriptomic differences. In addition, the systematically different interval between tissue collection and RNAlater preservation (< 1 min for PD biopsy specimens versus 1–20 min for surgically obtained controls) may represent a potential group-specific preanalytical confounder that cannot be fully separated from disease-related effects in the present study.. Finally, whole-blood transcriptomes are susceptible to variation arising from blood cell composition and haemolysis, and these variables were not available for the present cohort. In addition, one blood sample underwent RNA extraction after weekend storage at 4°C because the sample was collected on a Friday; although the sample met sequencing quality criteria and was retained, delayed processing may have contributed to its lower RNA integrity. These factors should be taken into account when interpreting the data generated using this protocol.

We anticipate that this protocol and accompanying dataset will serve as a valuable resource for future studies of peripheral transcriptomics in Parkinson’s disease, including biomarker discovery, cross-tissue transcriptomic comparisons, and the application of emerging transcriptomic technologies.

## Data Availability

The datasets presented in this study can be found in online repositories. The names of the repository/repositories and accession number(s) can be found at: https://www.ncbi.nlm.nih.gov/, PRJNA1027508.

## Footnotes

1
http://www.bioinformatics.babraham.ac.uk/projects/fastqc

2
http://www.usadellab.org/cms/?page=trimmomatic

3
https://daehwankimlab.github.io/hisat2/

4
https://ccb.jhu.edu/software/stringtie/

5
https://github.com/thelovelab/DESeq2

6
https://cutadapt.readthedocs.io/en/stable/

7
http://www.mirbase.org/

8
https://rnacentral.org/

9
https://www.mdc-berlin.de/content/mirdeep2-documentation

10
http://bowtie-bio.sourceforge.net/index.shtml
