# Supplementary material for: A validated workflow for paired total and small RNA sequencing from low-input submandibular gland biopsy specimens in de novo Parkinson’s disease patients
Source: Front Aging Neurosci. 2026 Aug 31;18:1931183. doi: 10.3389/fnagi.2026.1931183 (PMC13572653; doi:10.3389/fnagi.2026.1931183)
Supplement: Supplementary file 1 [file Data_Sheet_1.docx]

**Supplementary Table 1. Clinical and pathological characteristics of the neurologically unaffected surgical controls**

| Sample ID | Primary tumor | Stage * | Histology | CTx/RTx history ** | Pathologic  LN involvement |
| --- | --- | --- | --- | --- | --- |
| CTL_donor1 | Left tonsil | cT2N1M0 | HPV-positive  squamous cell carcinoma | No CTx; no RTx | No (0/50) |
| CTL_donor2 | Left tongue | T2N0/1 | Moderately differentiated  squamous cell carcinoma | Postoperative RTx to left tongue  and left neck levels I–III | No (0/33) |
| CTL_donor3 | Left parotid gland | Not documented | Secretory carcinoma | No CTx; no RTx | No (0/21) |
| CTL_donor4 | Tongue | T3N2c | Poorly differentiated  squamous cell carcinoma | No CTx; no RTx | Yes (3/78) |
| CTL_donor5 | Right tonsil | cT1N2M0 | HPV-positive  squamous cell carcinoma | Postoperative CCRT to right neck  levels II–IV (60 Gy) and right tonsillar  fossa (50–55 Gy) | Yes (4/43) |
| CTL_donor6 | Left tonsil | pT1N2M0 | HPV-associated  squamous cell carcinoma | Postoperative CCRT to left neck  and tonsillar bed (up to 62 Gy) | Yes (6/23) |

*Stage was reported as documented in the medical records. **Postoperative RT/CCRT was administered after surgical tissue collection where indicated.; CTx=Chemotherapy; RTx=Radiation therapy; LN=Lymph node; LN values are presented as the number of metastatic lymph nodes / total number of lymph nodes examined.

**Supplementary Table 2. Step-by-step protocol for RNA extraction from SMG tissue**

| Step | Procedure | Reagent/amount | Condition |
| --- | --- | --- | --- |
| 1. Tissue processing | Entire available SMG  tissue used | — | Tissue weight not measured |
| 2. Homogenization | Homogenize tissue | QIAzol, 750 μL;  3-mm bead | TissueLyser, 20 Hz, 2 min |
| 3. Phase separation | Add chloroform and  vortex | Chloroform, 150 μL | Vortex 15 s; RT 2–3 min |
| 4. Centrifugation | Separate aqueous phase | — | 12,000 ×g, 15 min, 4°C |
| 5. RNA precipitation | Add isopropanol | Equal volume of  100% isopropanol | RT 10 min |
| 6. Centrifugation | Pellet RNA | — | 12,000 ×g, 10 min, 4°C |
| 7. RNA wash | Wash pellet | 700 μL 70% ethanol | 12,000 ×g, 5 min, 4°C |
| 8. Final spin/drying | Remove residual ethanol | — | 12,000 ×g, 1 min, 4°C;  brief air-dry |
| 9. Resuspension | Resuspend RNA | 50 μL RNase-free water | — |
| 10. DNase | Not performed | — | — |
| 11. Storage | Store extracted RNA | — | ≤−80°C |
| 12. RNA QC | Assess integrity/quantity | RIN ≥7 recommended,  not exclusionary | Lower RIN accepted if library construction feasible |
| 13. Library input | Total RNA-seq | 0.5 μg/sample | Same across all PD and  control samples |
| 14. Library input | Small RNA-seq | 3 μg/sample | Same across all PD and  control samples |

**Supplementary Table 3. Small RNA-seq read counts before and after rRNA removal**

| Sample ID | Sample Type | SRA Run Accession No. | Total Read Count | Retained Read Count | Removed Read Count |
| --- | --- | --- | --- | --- | --- |
| PD_donor1 | SMG | SRR26372838 | 95,419,059 | 95,158,822 (99.73%) | 260,237 (0.27%) |
|  | Blood | SRR26372830 | 83,139,654 | 59,081,970 (71.06%) | 24,057,684 (28.94%) |
| PD_donor2 | SMG | SRR26372837 | 32,953,504 | 21,380,541 (64.88%) | 11,572,963 (35.12%) |
|  | Blood | SRR26372829 | 32,785,899 | 32,731,859 (99.84%) | 54,040 (0.16%) |
| PD_donor3 | SMG | SRR26372836 | 37,697,793 | 27,928,215 (74.08%) | 9,769,578 (25.92%) |
|  | Blood | SRR26372828 | 40,462,267 | 40,401,827 (99.85%) | 60,440 (0.15%) |
| PD_donor4 | SMG | SRR26372835 | 45,155,471 | 33,091,849 (73.28%) | 12,063,622 (26.72%) |
|  | Blood | SRR26372827 | 46,480,080 | 46,392,532 (99.81%) | 87,548 (0.19%) |
| PD_donor5 | SMG | SRR26372833 | 38,043,005 | 29,953,647 (78.74%) | 8,089,358 (21.26%) |
|  | Blood | SRR26372826 | 47,383,383 | 47,205,570 (99.62%) | 177,813 (0.38%) |
| PD_donor6 | SMG | SRR26372832 | 32,344,020 | 20,716,642 (64.05%) | 11,627,378 (35.95%) |
|  | Blood | SRR26372874 | 37,587,017 | 37,361,120 (99.4%) | 225,897 (0.6%) |
| PD_donor7 | SMG | SRR26372831 | 30,715,631 | 25,328,733 (82.46%) | 5,386,898 (17.54%) |
|  | Blood | SRR26372873 | 47,745,982 | 47,597,098 (99.69%) | 148,884 (0.31%) |
| CTL_donor1 | SMG | SRR26372871 | 36,690,959 | 10,682,834 (29.12%) | 26,008,125 (70.88%) |
|  | Blood | SRR26372865 | 51,607,534 | 47,667,047 (92.36%) | 3,940,487 (7.64%) |
| CTL_donor2 | SMG | SRR26372870 | 46,082,188 | 35,617,707 (77.29%) | 10,464,481 (22.71%) |
|  | Blood | SRR26372864 | 49,161,529 | 49,093,181 (99.86%) | 68,348 (0.14%) |
| CTL_donor3 | SMG | SRR26372869 | 34,797,232 | 27,622,546 (79.38%) | 7,174,686 (20.62%) |
|  | Blood | SRR26372863 | 46,901,310 | 46,610,885 (99.38%) | 290,425 (0.62%) |
| CTL_donor4 | SMG | SRR26372868 | 28,756,415 | 21,282,137 (74.01%) | 7,474,278 (25.99%) |
|  | Blood | SRR26372862 | 44,347,063 | 44,178,068 (99.62%) | 168,995 (0.38%) |
| CTL_donor5 | SMG | SRR26372867 | 42,630,181 | 38,687,506 (90.75%) | 3,942,675 (9.25%) |
|  | Blood | SRR26372860 | 44,406,244 | 44,012,433 (99.11%) | 393,811 (0.89%) |
| CTL_donor6 | SMG | SRR26372866 | 30,816,075 | 28,689,540 (93.1%) | 2,126,535 (6.9%) |
|  | Blood | SRR26372859 | 41,606,929 | 41,241,575 (99.12%) | 365,354 (0.88%) |

Total Read Count= Total Reads per sample (Trimmed read count + non-adapter read count); Retained Read Count= Reads remaining after rRNA removal; Removed Read Count= Reads removed by alignment to 45S pre-rRNA and mitochondrial rRNA

**Supplementary Table 4. Small RNA Genome Mapping Statistics**

| Sample ID | Sample Type | SRA Run Accession No. | Total Read Counts | Mapped Read | Unmapped Read |
| --- | --- | --- | --- | --- | --- |
| PD_donor1 | SMG | SRR26372838 | 59,081,970 | 56,457,651 (95.56%) | 2,624,319 (4.44%) |
|  | Blood | SRR26372830 | 95,158,822 | 91,571,981 (96.23%) | 3,586,841 (3.77%) |
| PD_donor2 | SMG | SRR26372837 | 21,380,541 | 19,316,039 (90.34%) | 2,064,502 (9.66%) |
|  | Blood | SRR26372829 | 32,731,859 | 32,382,519 (98.93%) | 349,340 (1.07%) |
| PD_donor3 | SMG | SRR26372836 | 27,928,215 | 26,571,907 (95.14%) | 1,356,308 (4.86%) |
|  | Blood | SRR26372828 | 40,401,827 | 39,918,353 (98.8%) | 483,474 (1.2%) |
| PD_donor4 | SMG | SRR26372835 | 33,091,849 | 31,455,386 (95.05%) | 1,636,463 (4.95%) |
|  | Blood | SRR26372827 | 46,392,532 | 45,996,074 (99.15%) | 396,458 (0.85%) |
| PD_donor5 | SMG | SRR26372833 | 29,953,647 | 28,597,534 (95.47%) | 1,356,113 (4.53%) |
|  | Blood | SRR26372826 | 47,205,570 | 45,268,034 (95.9%) | 1,937,536 (4.1%) |
| PD_donor6 | SMG | SRR26372832 | 20,716,642 | 19,409,469 (93.69%) | 1,307,173 (6.31%) |
|  | Blood | SRR26372874 | 37,361,120 | 36,028,636 (96.43%) | 1,332,484 (3.57%) |
| PD_donor7 | SMG | SRR26372831 | 25,328,733 | 24,500,300 (96.73%) | 828,433 (3.27%) |
|  | Blood | SRR26372873 | 47,597,098 | 46,854,800 (98.44%) | 742,298 (1.56%) |
| CTL_donor1 | SMG | SRR26372871 | 10,682,834 | 7,040,479 (65.9%) | 3,642,355 (34.1%) |
|  | Blood | SRR26372865 | 47,667,047 | 41,820,882 (87.74%) | 5,846,165 (12.26%) |
| CTL_donor2 | SMG | SRR26372870 | 35,617,707 | 34,232,766 (96.11%) | 1,384,941 (3.89%) |
|  | Blood | SRR26372864 | 49,093,181 | 48,570,305 (98.93%) | 522,876 (1.07%) |
| CTL_donor3 | SMG | SRR26372869 | 27,622,546 | 26,326,688 (95.31%) | 1,295,858 (4.69%) |
|  | Blood | SRR26372863 | 46,610,885 | 43,910,440 (94.21%) | 2,700,445 (5.79%) |
| CTL_donor4 | SMG | SRR26372868 | 21,282,137 | 20,520,263 (96.42%) | 761,874 (3.58%) |
|  | Blood | SRR26372862 | 44,178,068 | 43,303,961 (98.02%) | 874,107 (1.98%) |
| CTL_donor5 | SMG | SRR26372867 | 38,687,506 | 37,614,592 (97.23%) | 1,072,914 (2.77%) |
|  | Blood | SRR26372860 | 44,012,433 | 41,102,854 (93.39%) | 2,909,579 (6.61%) |
| CTL_donor6 | SMG | SRR26372866 | 28,689,540 | 27,891,807 (97.22%) | 797,733 (2.78%) |
|  | Blood | SRR26372859 | 41,241,575 | 37,122,386 (90.01%) | 4,119,189 (9.99%) |

Mapping statistics for processed small RNA reads aligned to the human reference genome (GRCh38) using Bowtie as part of the miRDeep2 workflow. Genome mapping was used to identify the genomic origin of small RNA reads and to define candidate loci for known and novel miRNA discovery.

**Supplementary Table 5. Small RNA Hairpin Mapping Statistics**

| Sample ID | Sample Type | SRA Run Accession No. | Total Read Counts | Mapped Read | Unmapped Read |
| --- | --- | --- | --- | --- | --- |
| PD_donor1 | SMG | SRR26372838 | 59,081,970 | 53,372,665 (90.34%) | 5,709,305 (9.66%) |
|  | Blood | SRR26372830 | 95,158,822 | 72,775,028 (76.48%) | 22,383,794 (23.52%) |
| PD_donor2 | SMG | SRR26372837 | 21,380,541 | 17,757,697 (83.06%) | 3,622,844 (16.94%) |
|  | Blood | SRR26372829 | 32,731,859 | 31,393,215 (95.91%) | 1,338,644 (4.09%) |
| PD_donor3 | SMG | SRR26372836 | 27,928,215 | 25,069,452 (89.76%) | 2,858,763 (10.24%) |
|  | Blood | SRR26372828 | 40,401,827 | 39,301,583 (97.28%) | 1,100,244 (2.72%) |
| PD_donor4 | SMG | SRR26372835 | 33,091,849 | 29,541,742 (89.27%) | 3,550,107 (10.73%) |
|  | Blood | SRR26372827 | 46,392,532 | 45,269,331 (97.58%) | 1,123,201 (2.42%) |
| PD_donor5 | SMG | SRR26372833 | 29,953,647 | 27,315,629 (91.19%) | 2,638,018 (8.81%) |
|  | Blood | SRR26372826 | 47,205,570 | 44,032,408 (93.28%) | 3,173,162 (6.72%) |
| PD_donor6 | SMG | SRR26372832 | 20,716,642 | 17,995,824 (86.87%) | 2,720,818 (13.13%) |
|  | Blood | SRR26372874 | 37,361,120 | 35,478,694 (94.96%) | 1,882,426 (5.04%) |
| PD_donor7 | SMG | SRR26372831 | 25,328,733 | 23,492,466 (92.75%) | 1,836,267 (7.25%) |
|  | Blood | SRR26372873 | 47,597,098 | 45,816,127 (96.26%) | 1,780,971 (3.74%) |
| CTL_donor1 | SMG | SRR26372871 | 10,682,834 | 3,482,437 (32.6%) | 7,200,397 (67.4%) |
|  | Blood | SRR26372865 | 47,667,047 | 38,587,181 (80.95%) | 9,079,866 (19.05%) |
| CTL_donor2 | SMG | SRR26372870 | 35,617,707 | 31,831,987 (89.37%) | 3,785,720 (10.63%) |
|  | Blood | SRR26372864 | 49,093,181 | 47,615,549 (96.99%) | 1,477,632 (3.01%) |
| CTL_donor3 | SMG | SRR26372869 | 27,622,546 | 25,008,249 (90.54%) | 2,614,297 (9.46%) |
|  | Blood | SRR26372863 | 46,610,885 | 43,195,330 (92.67%) | 3,415,555 (7.33%) |
| CTL_donor4 | SMG | SRR26372868 | 21,282,137 | 19,325,524 (90.81%) | 1,956,613 (9.19%) |
|  | Blood | SRR26372862 | 44,178,068 | 42,491,138 (96.18%) | 1,686,930 (3.82%) |
| CTL_donor5 | SMG | SRR26372867 | 38,687,506 | 36,266,027 (93.74%) | 2,421,479 (6.26%) |
|  | Blood | SRR26372860 | 44,012,433 | 40,356,195 (91.69%) | 3,656,238 (8.31%) |
| CTL_donor6 | SMG | SRR26372866 | 28,689,540 | 26,905,824 (93.78%) | 1,783,716 (6.22%) |
|  | Blood | SRR26372859 | 41,241,575 | 36,213,134 (87.81%) | 5,028,441 (12.19%) |

Mapping statistics for processed small RNA reads aligned to precursor miRNA (hairpin) sequences using miRDeep2. Hairpin mapping was used to assess compatibility of reads with known or predicted miRNA precursor structures and to support the identification of known and novel miRNAs.

**Supplementary Table 6. RNA categories in RNAcentral Database for species**

| Taxon ID | Species | Small RNA category |
| --- | --- | --- |
| 9606 | Homo sapiens | rRNA, tRNA, piRNA, snoRNA, snRNA, Y_RNA, scRNA, vault_RNA |
|  |  |  |

**Supplementary Table 7. Priority of small RNA category**

| Priority | Small RNA category |
| --- | --- |
| 1 | Known miRNA |
| 2 | Novel miRNA |
| 3 | piRNA |
| 4 | snoRNA |
| 5 | snRNA |
| 6 | rRNA |
| 7 | tRNA |
| 8 | Y RNA |
| 9 | scRNA |
| 10 | vault RNA |
| 11 | Genome |
| 12 | Repeat |
| 13 | Mycoplasma |
| 14 | TruSeqIndexedAdapter |
